# Cardiac myofibroblasts: cells out of balance. A new thematic series

**DOI:** 10.1186/1755-1536-5-14

**Published:** 2012-09-03

**Authors:** Ian M C Dixon

**Affiliations:** 1 Principal Investigator, Laboratory of Molecular Cardiology, Department of Physiology, Faculty of Medicine and the Institute of Cardiovascular Sciences, St. Boniface General Hospital Research Centre, University of Manitoba, 351 Tache Ave, Manitoba, R2H 2A6, Winnipeg, Canada

**Keywords:** Cardiac fibrosis, Cardiac fibroblasts, Myofibroblasts, Phenoconversion, Extracellular matrix

## Abstract

We are pleased to introduce a new thematic series dealing with cardiac fibrosis and its association with cardiovascular diseases. A wide variety of cardiovascular diseases are associated with cardiac fibrosis, which is now widely recognized to be not a secondary, but rather a primary contributor to cardiac dysfunction. The purpose of the current series of papers and reviews is to provide the reader with an up-to-date synopsis of the very latest research results and hypotheses that impact on cardiac fibrosis and disease.

## 

*Koyaanisqatsi* is a Hopi word meaning “life out of balance” and is the title of the popular environmentalist movie shot nearly 2 decades ago. The film contrasts speeded-up images of American desert landscapes and life without man to smokestacks and expressways and an endless flow of humanity through the streets of any large city. The movie is beautifully created and supported by technical excellence in imagery and sound, to the point where one might make an honest mistake and misinterpret the main message, as indeed some of the images that were intended to repel to the viewer are appealing themselves. Using this representation of despoiled environment as an allegory of the presence of fibroblasts and their role in pathogenesis of fibrotic heart disease, the connection may be more easily discerned. In this regard, our laboratory has used an *in vitro* scratch assay and has compiled data depicting a greatly accelerated account of a 16-h duration showing migration of activated myofibroblasts (Additional file 1: Figure 1). Others have aptly described this assay as a “heart attack in a plate,” which is a convenient and inexpensive method to assess myofibroblast migration from the highly confluent area of cells into the denuded region [1]. In this figure, virtually all of the cells in the area photographed are myofibroblasts. Studying the film loop of myofibroblasts reveals a number of interesting phenomena. Cells in proximity to the denuded region become suddenly motile, and they reorient, divide, and migrate at greatly accelerated velocity *vs.* their relatively quiescent neighbors. While their movement from an area of relative confluence to the denuded area is fascinating in itself, it is likely a reasonably accurate reflection of their motility *in vivo,* e.g., in ongoing wound healing of necrosed muscle in post-MI heart and/or stroma. We have previously shown that cardiac myofibroblasts are hypersynthetic and are key players in the development of cardiac fibrosis. As such, they are included within the focus of a new thematic series of reviews (Cardiovascular Diseases) to provide information about some of the recent developments in our understanding of the pathogenesis of cardiac fibrosis, to be published during the coming year in *Fibrogenesis and Tissue Repair*.

What is the difference between healthy cardiac fibroblast function and that of cardiac myofibroblasts? Matrix components of the healthy heart are produced by interstitial cardiac fibroblasts [2]. These cells maintain a relatively slow turnover of fibrillar collagens [3] in normal conditions, but may respond to both mechanical loading [4] and TGF-β1 stimulation [5,6] by a switch to a myofibroblastic phenotype wherein they express α-smooth muscle actin (αSMA) [7], synonymous with increased contractile force [8]. αSMA expression is increased in myofibroblasts in fibrotic hearts subjected to pressure or volume overload or in the infarct scar of post-MI hearts [3]. Causal factors in this conversion are compressibility of the substrate when ventricular fibroblasts are plated *in vitro*[9] and overexpression of R-Smads [10]. Enhanced contractility that attends this protein’s expression is believed to be important in allowing these cells to contract while bound to matrix collagens and other proteins, thereby allowing for physical remodeling of the matrix itself [11]. Thus myofibroblasts are the primary mediators of wound healing in the damaged ventricle and we have previously demonstrated that they are the dominant cell type in the infarct scar [12]. Myofibroblasts migrate to the infarct zone, restoring cellularity [13]. Their contraction confers matrix remodeling by imparting tensile force to the matrix, opposes retractile force, promotes scar contraction, activates latent TGF-β1, and reorients collagen fibrils [4,14-16]. Investigation of these cells in hypertrophied hearts is clinically relevant as they contribute to wound healing, matrix remodeling, and eventual cardiac fibrosis through the elevated production of fibrillar and non-fibrillar collagens [17,18].

The new thematic series to be published in this journal will touch upon topics not commonly associated with “textbook” knowledge of cardiac fibroblasts. Included in the range of topics in which we will be accepting submissions of novel research papers and review articles are: (1) the role of fibroblast and myofibroblast autophagy in the regulation of cellular function, (2) the relative contribution of migratory stem cells in populating the heart with myofibroblasts *vs.* endogenous conversion of fibroblasts to myofibroblasts, (3) the roles of pericytes, epithelial-to-mesenchymal transition (EMT), endothelial-to-mesenchymal transition (EndMT), and fibrocytes in influencing cardiac extracellular matrix remodeling in heart disease, (4) microRNA influence on myofibroblast function, (5) novel signaling pathways and mechanotransduction in the regulation of myofibroblast function, (6) exploration of the role of non-collagenous proteins in the cardiac extracellular matrix, (7) developments in MMP and TIMP biology in the heart, (8) valvular interstitial cells and valve disease, and finally, (9) novel aspects of myofibroblast biology. As these areas represent novel concepts in heart disease and cardiac fibrosis and could be described as areas less well-covered in the literature, they are to be our current focus.

Why should clinicians or basic scientists care about these new developments? Part of the answer lies in the novel suggestion that cardiac fibroblasts and myofibroblasts are unique cells, e.g., distinguished by their relative specific phenotype from other fibroblastic cells. While it is well-known that the structural scaffold that exists between myocytes in the heart is composed of extracellular matrix (matrix) and mesenchymal cells and that this matrix provides an exceptionally strong means to tether and coordinate force generated via myocyte contraction, little is known of their regulation. The principal stromal cell type is fibroblasts, but this designation belies their diversity and topographic differentiation from organ to organ including the heart [19]. Thus, the term “fibroblasts” designates a highly heterogeneous group that exhibits distinct differentiated phenotypes in different tissues [19]. The implications of these fundamental differences are unclear. Further, the study of fibroblast and myofibroblast biology in specific organs is an important but relatively understudied area, especially in cardiovascular disease with attendant cardiac fibrosis. Recent novel data indicate that ventricular fibroblast activation and cardiac fibrosis are primary events in ventricular remodeling, rather than a secondary response to cardiomyocyte injury [20]. Thus the traditional role of cardiac fibrosis as a secondary disease modifier has, for the first time, been called into question [20], and the need to investigate factors that regulate cardiac myofibroblasts in various types of cardiovascular disease is apparent. For these reasons we have sought to publish a series of papers that will highlight the molecular and biochemical properties of these specific cells, and of their functioning in disease. Finally, the field of cardiac fibrosis has experienced a burgeoning level of interest and participation in investigations during the past 5 years, and this series will take advantage of this to showcase the thoughts of what some of the best minds in area are thinking.

## Competing interests

The authors declare that they have no competing interests.

## Supplementary Material

Additional file 1 **Figure 1** Scratch Assay of Cardiac Myofibroblasts. Time-lapse movie (10 s) of primary rat cardiac myofibroblasts in first passage compressed form 16 hours of incubation in a Zeiss live-cell incubation in a Zeiss live-cel l incubation chamber. Cells are cultured in DMEM/F 12 in the presence of fetal bovine serum. These cells express significant levels of a SMA and ED-A fibronectin proteins that designate them as myofibroblasts. Click here for file
